# Myocardial injury detected by T1 and T2 mapping on CMR predicts subsequent cancer therapy–related cardiac dysfunction in patients with breast cancer treated by epirubicin-based chemotherapy or left-sided RT

**DOI:** 10.1007/s00330-021-08260-7

**Published:** 2021-09-18

**Authors:** Enver Tahir, Manuella Azar, Sahar Shihada, Katharina Seiffert, Yvonne Goy, Antonia Beitzen-Heineke, Isabel Molwitz, Kai Muellerleile, Christian Stehning, Gerhard Schön, Gerhard Adam, Cordula Petersen, Volkmar Müller, Gunnar K. Lund

**Affiliations:** 1 Department of Diagnostic and Interventional Radiology and Nuclear Medicine, University Hospital Hamburg Eppendorf, Martinistr. 52, 20246 Hamburg, Germany; 2 Department of Gynecology, University Hospital Hamburg-Eppendorf, Hamburg, Germany; 3 Department of Radiotherapy and Radio-Oncology, University Hospital Hamburg Eppendorf, Hamburg, Germany; 4 Department of Oncology, Hematology and Bone Marrow Transplantation With the Section Pneumology, University Hospital Hamburg Eppendorf, Hamburg, Germany; 5 Department of General and Interventional Cardiology, University Heart Center, Hamburg, Germany; 6 Philips Clinical Science, Hamburg, Germany; 7 Department of Medical Biometry and Epidemiology, University Hospital Hamburg-Eppendorf, Hamburg, Germany

**Keywords:** Breast cancer, Chemotherapy, Radiation therapy, Magnetic resonance, Heart

## Abstract

**Objectives:**

Cancer therapy-related cardiac dysfunction (CTRCD) is a relevant clinical problem and needs early prediction. This study aimed to analyze myocardial injury using serial laboratory and cardiac magnetic resonance imaging (CMR) parameters after epirubicin-based chemotherapy compared with left-sided radiotherapy and to study their value for early prediction of CTRCD.

**Methods:**

Sixty-six consecutive women (53 ± 13 years) including *n* = 39 with epirubicin-based chemotherapy and n = 27 with left-sided radiotherapy were prospectively studied by 3 T CMR including left ventricular (LV) mass and volumes for ejection fraction (LVEF), as well as feature-tracking with global longitudinal strain (GLS) and T1/T2 mapping. CMR was performed at baseline, at therapy completion (follow-up 1, FU1), and after 13 ± 2 months (FU2). CTRCD was defined as LVEF decline of at least 10% to < 55% or a > 15% GLS change at FU2.

**Results:**

T1 and T2 increased at FU1 after epirubicin-based chemotherapy, but not after left-sided radiotherapy. CTRCD occurred in 20% of patients after epirubicin-based chemotherapy and in 4% after left-sided radiotherapy. T1 at FU1 was the best single parameter to predict CTRCD with an area under the curve (AUC) of 0.712 (CI 0.587–0.816, *p* = 0.005) with excellent sensitivity (100%, 66–100%), but low specificity (44%, 31–58%). Combined use of increased T1 and LVEF ≤ 60% at FU1 improved AUC to 0.810 (0.695–0.896) resulting in good sensitivity (78%, 44–95%) and specificity (84%, 72–92%).

**Conclusion:**

Only epirubicin-based chemotherapy, but not left-sided radiotherapy, resulted in increased T1/T2 myocardial relaxation times as a marker of myocardial injury. Combined use of CMR parameters may allow an early prediction of subsequent CTCRD.

**Key Points:**

• *Myocardial T1 and T2 relaxation times increased at FU1 after epirubicin-based chemotherapy, but not after left-sided radiotherapy.*

• *Cancer therapy–related cardiac dysfunction (CTRCD) occurred in 20% of patients after epirubicin-based chemotherapy and in 4% after left-sided radiotherapy.*

• *Combined use of increased T1 and reduced LVEF had an AUC of 0.810 (0.695–0.896) to predict CTRCD with good sensitivity (78%, 44–95%) and specificity (84%, 72–92%).*

**Supplementary Information:**

The online version contains supplementary material available at 10.1007/s00330-021-08260-7.

## Introduction

The standard of care in early breast cancer, which has not spread beyond the breast or the axillary lymph nodes, includes systemic anthracycline-based chemotherapy and/or radiotherapy [1, 2]. A typical scenario for chemotherapy followed by postoperative radiotherapy would be a tumor > 2 cm or not feasible for optimal surgery and wish for breast conservation as recommended by current guidelines [2]. Epirubicin and doxorubicin are the most widely used anthracyclines for breast cancer treatment but are associated with increased risk for cancer therapy–related cardiac dysfunction (CTRCD) [3]. Radiotherapy of breast cancer is strongly recommended after breast-conserving therapy both in combination with preceding chemotherapy or radiotherapy alone [2]. However, radiotherapy resulted in long-term cardiac toxicity such as heart failure and myocardial infarction related to heart radiation [4, 5]. Early studies showed that the risk for cardiovascular death was increased in patients with left-sided breast cancer as a result of higher cardiac radiation [4, 5]. The cardiotoxic effect was presumably mediated by the progression of coronary heart disease; however, the aforementioned studies were performed several decades ago by using less well-established dose reduction strategies. The cardiotoxic effect of current radiation strategies with reduced cardiac dose such as three-dimensional CT-based planning and deep inspiration breath-hold technique are less well understood.

Cardiac magnetic resonance imaging (CMR) allows accurate detection of myocardial injury using novel T1 and T2 mapping techniques, which detect myocardial edema related to acute injury [6, 7]. A recent animal study by Galán-Arriola et al underscored their value for detection of cardiotoxicity after intracoronary injection of doxorubicin [8]. This study revealed that T2 was the earliest mapping parameter of cardiotoxicity. T2 increased 6 weeks after chemotherapy initiation and correlated with intracardiomyocyte edema. Subsequently, T1 increased and was associated with the decline of LV ejection fraction [8]. We hypothesized that T1/T2 mapping CMR is able to detect cardiotoxicity in patients either treated by epirubicin-based chemotherapy or by radiotherapy and is useful for early prediction of subsequent CTRCD.

The aims to prospectively analyze myocardial injury using serial T1/T2 mapping CMR and laboratory parameters after epirubicin-based chemotherapy compared with left-sided radiotherapy and to study the value of these parameters for early prediction of CTRCD.

## Materials and methods

### Patient groups and CMR scheduling

**Fig. 1:**
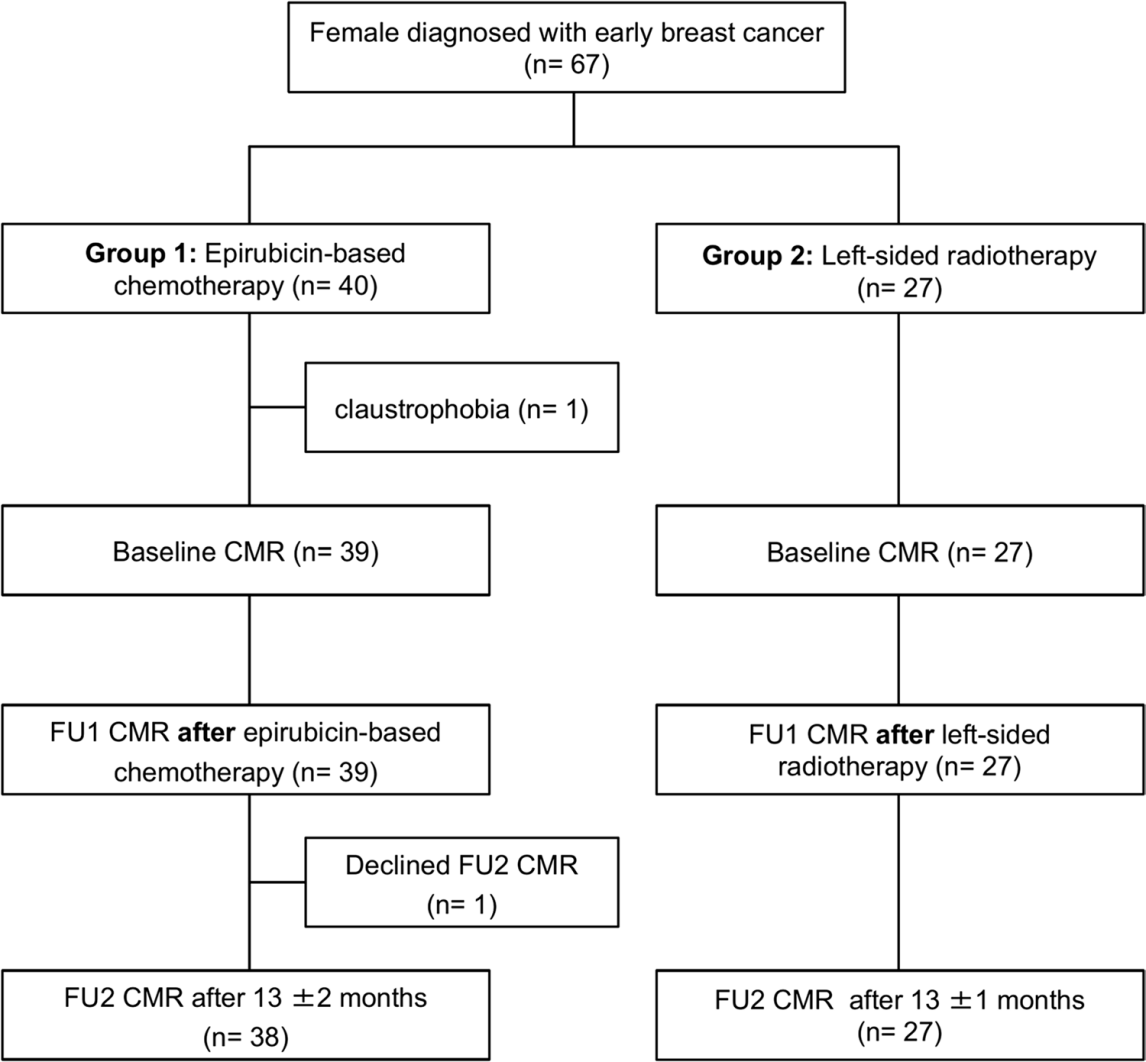
Patient flow chart

**Fig. 2:**
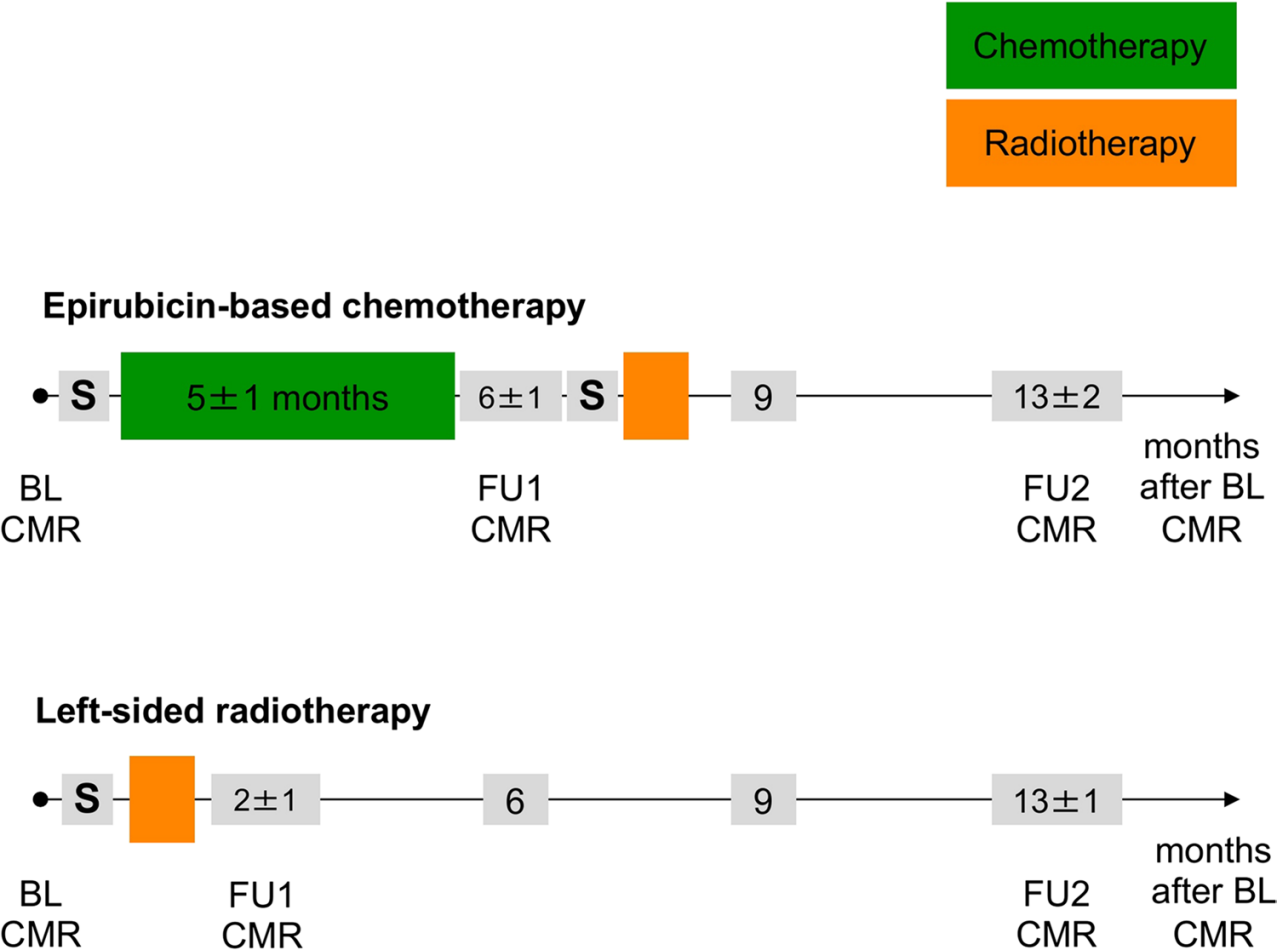
Treatment timeline of patients with breast cancer. Patients with epirubicin-based chemotherapy received therapy for 5 ± 1 months followed by follow-up 1 (FU1) CMR at 2 ± 2 weeks after completion of therapy. Surgery (**S**) was either performed before (*n* = 15) or after chemotherapy (*n* = 24). Patients with left-sided radiotherapy received initial surgery, followed by radiotherapy and FU1 CMR at 1 ± 2 weeks after completion of radiotherapy. Follow-up 2 (FU2) CMR was performed in both groups at 13 months after baseline (BL) CMR

The local ethics committee approved the study (PV5292) and all women gave their written informed consent. Women with breast cancer were prospectively recruited between October 2016 and October 2017 (Fig. 1). The treatment strategy was decided by an interdisciplinary tumor board as part of clinical routine. Subsequently, patients were either treated with epirubicin-based chemotherapy followed by radiotherapy (group 1) or with left-sided radiotherapy (group 2). Patients were not randomized to the treatment strategies of group 1 or group 2. Group 1 included 39 patients (age: 51 ± 11 years, range 30–75 years) and one subject was excluded due to claustrophobia at baseline CMR (Fig. 1). Chemotherapy consisted of 4 cycles of epirubicin (90 mg/m^2^) combined with cyclophosphamide (600 mg/m^2^) and 12 cycles of paclitaxel (80 mg/m^2^) according to current guidelines [2]. Five group 1 patients (13%) additionally received trastuzumab due to human epidermal growth factor receptor 2 positive (HER2 +) status. Chemotherapy lasted for 5 ± 1 months and follow-up 1 (FU1) CMR was performed at 2 ± 2 weeks after completion (Fig. 2). Subsequently, 18 group 1 patients (46%) underwent left-sided radiotherapy, whereas 21 (54%) obtained right-sided radiotherapy. Surgery was either performed before (*n* = 15, 38%) or after chemotherapy (*n* = 24, 62%). FU2 CMR was acquired in group 1 at 13 ± 2 months after BL CMR.

Group 2 included 27 women (age: 56 ± 14 years, range 28–82 years) with left-sided breast cancer, who were treated with initial surgery followed by left-sided radiotherapy lasting for 1.2 ± 0.4 months (Fig. 2). Whole breast radiation was performed using a linear accelerator (Varian TrueBeam®, Varian Medical Systems). Three-dimensional tangential treatment plans with a “field-in-field” technique were established to minimize the heart dose [9]. A deep inspiration breath-hold technique was applied to reduce the heart radiation dose. Respiratory motion was monitored using the Varian respiratory gating system and the patient was instructed to take a deep breath in and hold it. The treatment beam was stopped when the breathing signal dropped outside a defined threshold for the respiratory motion of the heart [10]. The total whole-breast radiation dose was 48 ± 4 Gy and the mean cardiac dose was 2 ± 2 Gy. FU1 CMR was acquired at 1 ± 2 weeks after completion and FU2 CMR was obtained 13 ± 1 months after BL CMR. Blood samples were drawn immediately before each CMR.

### CMR protocol

CMR was performed on a 3.0-T scanner (Ingenia, Philips Medical Systems). The CMR protocol included a cine short-axis stack for LV volumes, mass, and function assessment using a standard steady-state free-precession (SSFP) sequence. T2 mapping was performed using a free-breathing navigator-gated black-blood prepared gradient and spin-echo (GraSE) hybrid sequence in three short-axis slices (basal, midventricular, and apical) [11]. T1 mapping was performed using a 5 s(3 s)3 s MOLLI sequence with typical imaging parameters: voxel size 2 × 2 × 10 mm^3^, echo time = 0.7 ms, time to repetition = 2.3 ms, partial echo factor = 0.8, flip angle = 35°, SENSE factor = 2, linear phase encoding, ten start-up cycles to approach steady-state prior to imaging, typical effective inversion times between 134 and 5500 ms [16]. Ten minutes after injection of 0.15 mmol/kg gadoterate meglumine (Dotarem®, Guerbet), end-diastolic late gadolinium enhancement (LGE) images were acquired using phase-sensitive inversion recovery (PSIR) sequences in short-axis and two-, three-, and four-chamber views [12]. Details are in Supplemental Material.

### CMR data analysis

Two investigators (M.A. and S.S.) independently and blindly analyzed each CMR in random order using CVi42 software (Circle Cardiovascular Imaging Inc). Parameters were indexed to the body surface area (BSA). Evaluation of LV and right ventricular (RV) volumes and LV mass was performed in standard fashion [13]. To measure global native T1 and T2 relaxation times and ECV, corresponding short-axis maps were used to carefully delineate endo- and epicardial contours with 10% endo- and epicardial offsets to avoid contamination. The presence of LGE was visually analyzed [13].

### Myocardial strain analysis

Myocardial strain was analyzed on cine CMR images using Segment feature-tracking software version 2.1.R.6108 (Medviso) [14]. In short, this software analyzes myocardial strain by computing interframe deformation fields using an endocardial tracking strategy based on non-rigid image registration [14]. Global peak systolic LV longitudinal (GLS) and radial strain (GRS) were measured on 3 long-axis cine series, whereas circumferential strain (GCS) was measured on three short-axis cine series. Endo- and epicardial contours were manually delineated on end-diastolic images and were then automatically propagated by the software throughout the cardiac cycle generating myocardial strain [14].

### Cancer therapy-related cardiac dysfunction

Presence of cancer therapy–related cardiac dysfunction (CTRCD) was defined as a decline in LVEF of at least 10 to < 55% or a > 15% GLS change at FU2 CMR [3, 15].

### Statistical analysis

Statistical analysis was performed using MedCalc for Windows, version 13.3.3.0 (MedCalc Software), and SPSS for Windows, version 21.0 (IBM SPSS Statistics). All CMR data are given as the mean of two observers. Continuous data are presented as mean ± SD and categorical data are presented as absolute numbers and percentages. A mixed model was applied to analyze changes in the obtained parameters with time and pairwise comparison. A receiver-operating characteristic (ROC) analysis was performed to evaluate the performance of the obtained values at FU1 to predict the occurrence of CTRCD at FU2 by calculating areas under the curve (AUC) and optimal cut-off values from the ROC curves using the Youden-index. Statistical significance was defined as *p* < 0.05.

## Results

### Baseline demographics of both patient groups

Patients with epirubicin-based chemotherapy had higher weight, body mass index (BMI), and body surface area (BSA) compared to patients with left-sided radiotherapy (Table 1). Cancer stage is given for both groups in Table 1. There were no differences regarding cardiovascular risk factors and medication. No patient had known heart failure. High sensitive troponin T and NT-proBNP revealed no cardiac injury at baseline (Table 1).

**Table 1:** Baseline demographics of both patient groups

	Patients with epirubicin-based chemotherapy (*n* = 39)	Patients with left-sided radiotherapy (*n* = 27)	*p* value
Demographics			
Age, yrs	51 ± 11	56 ± 14	0.08
Weight, kg	77 ± 15	69 ± 9	0.02
Height, m	1.68 ± 0.07	1.68 ± 0.07	0.72
BMI, kg/m^2^	27 ± 5	25 ± 3	0.03
BSA, m^2^	1.86 ± 0.18	1.78 ± 0.13	0.04
Cancer stage			
DCIS, *n* (%)	0 (0)	4 (15)	0.024
T1, *n* (%)	8 (21)	17 (63)	0.028
T2, *n* (%)	25 (64)	6 (22)	0.001
T3, *n* (%)	2 (5)	0 (0)	0.51
T4, *n* (%)	4 (10)	0 (0)	0.14
N0, *n* (%)	18 (46)	24 (89)	< 0.001
N1, *n* (%)	21 (54)	2 (7)	< 0.0001
N2, *n* (%)	0 (0)	1 (4)	0.41
N3, *n* (%)	0 (0)	0 (0)	> 0.99
M0, *n* (%)	39 (100)	27 (100)	> 0.99
M1, *n* (%)	0 (0)	0 (0)	> 0.99
Cardiovascular risk factors			
Hypertension, *n* (%)	6 (15)	10 (37)	0.08
Diabetes, *n* (%)	0 (0)	1 (4)	0.41
Dyslipidemia, *n* (%)	1 (3)	2 (7)	0.56
Current smoking, *n* (%)	4 (10)	1 (4)	0.64
Family history of CAD, *n* (%)	3 (8)	2 (7)	> 0.99
Known CAD, *n* (%)	1 (3)	1 (4)	> 0.99
Cardiac medication			
Aspirin/clopidogrel, *n* (%)	1 (3)	3 (11)	0.30
Statins, *n* (%)	1 (3)	2 (7)	0.56
Beta-blockers, *n* (%)	4 (10)	3 (11)	> 0.99
ACEI or ARB, *n* (%)	6 (15)	6 (22)	0.53
Laboratory tests			
HS TNT, pg/ml	5 ± 4	5 ± 2	0.68
NT-proBNP, pg/ml	121 ± 118	98 ± 85	0.40
Hematocrit, %	37 ± 4	37 ± 3	0.96
Hb, mg/dl	12.5 ± 1.1	12.7 ± 1.0	0.54
Creatinine, mg/dl	0.82 ± 0.2	0.77 ± 0.12	0.20

Abbreviations: *ACEI*, angiotensin-converting enzyme inhibitor; *ARB*, angiotensin receptor blocker; *BMI*, body mass index; *BSA*, body surface area; *CAD*, coronary artery disease; *Hb*, hemoglobin; *HS TNT*, high sensitive troponin T; *NT-proBNP*, N-terminal pro-brain natriuretic peptide

### Therapy-induced changes of laboratory and CMR parameters after chemotherapy

**Fig. 3:**
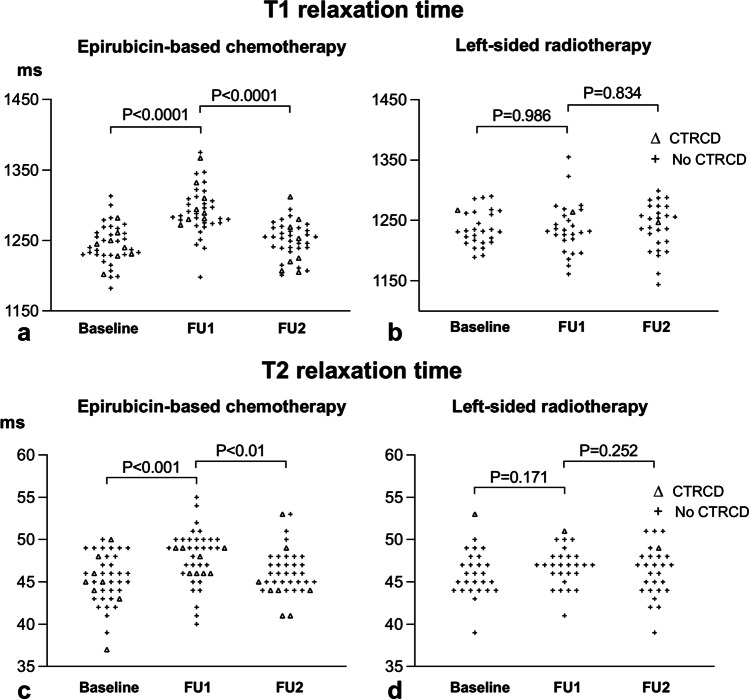
Development of T1 and T2 in patients with epirubicin-based chemotherapy (**a** and **c**) and left-sided radiotherapy (**b** and **d**). Epirubicin-based chemotherapy resulted in a homogeneous increase of T1 at follow-up 1 (FU1), whereas T1 was only increased at FU1 in two patients with left-sided radiotherapy, who did not develop cancer therapy-related cardiac dysfunction (CTRCD) at follow-up 2 (FU2)

High sensitive troponin T increased after epirubicin-based chemotherapy from 5 ± 4 to 8 ± 4 pg/ml (*p* = 0.04) and remained increased at FU2 with 8 ± 11 pg/ml (Table 2). NT-proBNP was unchanged from baseline to FU1 and FU2. Hematocrit and hemoglobin decreased at FU1 and then normalized at FU2. T1 and T2 increased at FU1 to 1293 ± 34 ms and 48 ± 3 ms respectively, compared to baseline with 1244 ± 29 ms (*p* < 0.001) and 45 ± 3 ms (*p* < 0.001) respectively, indicating the presence of myocardial edema (Table 2, Fig. 3). At FU2, T1 and T2 returned to baseline. Global longitudinal and circumferential strains were reduced at FU1 (*p* = 0.01 for GLS; *p* = 0.03 for GCS) and FU2 (*p* = 0.01 for both) with less negative strain values of − 17 ± 2% and − 17 ± 3% compared to baseline with − 18 ± 2% and − 18 ± 2%, respectively (Table 2), indicating reduced LV contractility. In contrast, LV ejection fraction was unchanged throughout (Table 2). LV, LA, and RV volumes decreased at FU1, indicating volume depletion following chemotherapy and LV and LA volumes normalized at FU2. No LGE was present at baseline and FU2, indicating the absence of focal myocardial fibrosis. ECV was normal at both time points with 28 ± 2% and 29 ± 2%, respectively (*p* = 0.52), excluding diffuse myocardial fibrosis. There was no indication that trastuzumab had additional cardiotoxic effects (Table S1). Laboratory and mapping parameters, ejection fraction, and myocardial strain in group 1 patients without (*n* = 34) and with trastuzumab treatment (*n* = 5) were similar at all times.

**Table 2:** Therapy-induced changes of laboratory and CMR parameters in patients with epirubicin-based chemotherapy

	Baseline (*n* = 39)	FU1 (*n* = 39)	FU2 (*n* = 38)	*p* value for FU1 vs. FU2
Laboratory tests				
HS TNT, pg/ml	5 ± 4	8 ± 4*	8 ± 11^ll^	0.99
NT-proBNP, pg/ml	121 ± 118	134 ± 274	142 ± 177	0.68
Hematocrit, %	37 ± 4	35 ± 4^‡^	38 ± 3	< 0.001
Hb, mg/dl	12.5 ± 1.1	11.6 ± 1.2^‡^	12.6 ± 1.0	< 0.001
Creatinine, mg/dl	0.82 ± 0.2	0.76 ± 0.14	0.83 ± 0.12	0.01
Mapping parameters				
T1, ms	1244 ± 29	1293 ± 34^‡^	1250 ± 26	< 0.001
T2, ms	45 ± 3	48 ± 3^‡^	46 ± 3	0.007
Extracellular volume (%)	28 ± 2		29 ± 2	
Global LV strain				
GLS, %	− 18 ± 2	− 17 ± 2*	− 17 ± 2^ll^	0.99
GCS, %	− 18 ± 2	− 17 ± 3*	− 17 ± 3^ll^	0.70
GRS, %	36 ± 7	34 ± 8	34 ± 6	0.92
Left heart parameters				
LVEF, %	60 ± 5	60 ± 6	60 ± 6	0.51
LV mass index, g/m^2^	51 ± 5	51 ± 7	52 ± 7	0.49
LVEDVi, ml/m^2^	76 ± 10	72 ± 13^†^	76 ± 12	0.003
LVESVi, ml/m^2^	30 ± 6	29 ± 7	31 ± 7	0.002
LVSVi, ml/m^2^	46 ± 7	43 ± 9*	45 ± 8	0.09
LAESVi, ml/m^2^	34 ± 10	30 ± 9^†^	34 ± 10	< 0.001
LAEDVi, ml/m^2^	15 ± 6	14 ± 4	15 ± 6	0.09
Right heart parameters				
RVEF, %	56 ± 7	58 ± 8	58 ± 6	0.59
RVEDVi, ml/m^2^	77 ± 11	73 ± 14^†^	74 ± 13	0.41
RVESVi, ml/m^2^	34 ± 8	31 ± 9^†^	31 ± 6^ll^	0.57
RVSVi, ml/ m^2^	43 ± 8	43 ± 9	43 ± 10	0.70
RAESVi, ml/m^2^	35 ± 9	30 ± 8^‡^	35 ± 10	< 0.001
RAEDVi, ml/m^2^	20 ± 6	16 ± 5^‡^	17 ± 7^¶^	0.14
Presence of LGE, *n* (%)	0 (0)	0 (0)	0 (0)	0 (0)

Numbers are mean ± SD for continuous and n (%) for categorical data

^*^p < 0.05, ^†^*p* < 0.01, or ^‡^*p* < 0.001 for baseline vs. FU1

^ll^*p* < 0.05 or ^¶^*p* < 0.01 for baseline vs. FU2

Abbreviations: *GCS*, global circumferential strain, *GLS*, global longitudinal strain, *GRS*, global radial strain; *Hb*, hemoglobin; *HS TNT*, high sensitive troponin T; *LA*, left atrial; *LAEDVi*, left atrial end-diastolic volume index; *LAESVi*, left atrial end-systolic volume index; *HS TNT*, high sensitive troponin T; *LGE*, late gadolinium enhancement; *LV*, left ventricular; *LVEF*, left ventricular ejection fraction; *LVEDVi*, left ventricular end-diastolic volume index; *LVESVi*, left ventricular end-systolic volume index; *LVSVi*, left ventricular stroke volume index; *NT-proBNP*, N-terminal pro-brain natriuretic peptide; *RA*, right atrial; *RAEDVi*, right atrial end-diastolic volume index; *RAESVi*, right atrial end-systolic volume index; *RV*, right ventricular; *RVEF*, right ventricular ejection fraction; *RVEDVi*, right ventricular end-diastolic volume index; *RVESVi*, right ventricular end-systolic volume index; *RVSVi*, right ventricular stroke volume index

### Therapy-induced changes of laboratory and CMR parameters after radiotherapy

High sensitive troponin T slightly increased after left-sided radiotherapy to 6 ± 3 pg/ml at FU1 (*p* < 0.04) and normalized at FU2 (Table 3). All other laboratory parameters were unchanged at FU1 and FU2. Mean T1 and T2 remained constant at FU1, indicating the absence of myocardial edema (Table 3, Fig. 3). This was paralleled by normal LV function measured by ejection fraction and myocardial strain (Table 3). A slight reduction in LV and RV volumes was observed at FU1 and FU2. No focal or diffuse myocardial fibrosis was observed by LGE or ECV.

**Table 3:** Therapy-induced changes of laboratory and CMR parameters with left-sided radiotherapy

	Baseline (*n* = 27)	FU1 (*n* = 27)	FU2 (*n* = 27)	*p* value for FU1 vs. FU2
Laboratory tests				
HS TNT, pg/ml	5 ± 2	6 ± 3*	5 ± 2	0.04
NT-proBNP, pg/ml	98 ± 85	91 ± 73	107 ± 118	0.21
Hematocrit, %	37 ± 3	38 ± 2	38 ± 2	0.62
Hb, mg/dl	12.7 ± 1.0	12.9 ± 0.8	12.7 ± 0.8	0.63
Creatinine, mg/dl	0.77 ± 0.12	0.77 ± 0.09	0.78 ± 0.11	0.56
Mapping				
T1, ms	1237 ± 29	1237 ± 42	1239 ± 39	0.80
T2, ms	46 ± 3	47 ± 2	46 ± 3	0.30
Extracellular volume (%)	30 ± 3		30 ± 3	
Global LV strain				
GLS, %	-18 ± 2	-18 ± 2	-18 ± 1	0.33
GCS, %	-18 ± 2	-18 ± 2	-19 ± 3	0.11
GRS, %	39 ± 6	39 ± 9	39 ± 7	0.90
CMR – left heart				
LVEF, %	62 ± 5	64 ± 6	62 ± 5	0.19
LV mass index, g/m^2^	51 ± 5	52 ± 6	52 ± 6	0.59
LVEDVi, ml/m^2^	78 ± 10	75 ± 11*	72 ± 11^*§*^	0.06
LVESVi, ml/m^2^	30 ± 6	29 ± 11	27 ± 6	0.34
LVSVi, ml/m^2^	48 ± 6	48 ± 10	45 ± 7^‡^	0.05
LAESVi, ml/m^2^	36 ± 10	34 ± 10	33 ± 10	0.54
LAEDVi, ml/m^2^	16 ± 7	15 ± 7	15 ± 6	0.89
CMR – right heart				
RVEF, %	57 ± 6	58 ± 7	60 ± 7	0.22
RVEDVi, ml/m^2^	81 ± 11	75 ± 14*	75 ± 15^‡^	0.99
RVESVi, ml/m^2^	36 ± 7	32 ± 7^†^	30 ± 8^*§*^	0.28
RVSVi, ml/ m^2^	45 ± 7	44 ± 10	45 ± 11	0.50
RAESVi, ml/m^2^	38 ± 12	37 ± 13	38 ± 15	0.61
RAEDVi, ml/m^2^	21 ± 7	19 ± 8	19 ± 11	0.94
LGE lesions, n (%)	0 (0)	0 (0)	0 (0)	0 (0)

Numbers are mean ± SD for continuous and *n* (%) for categorical data

^*^p < 0.05 or ^†^*p* < 0.01 for baseline vs. FU1

^‡^*p* < 0.05 or ^*§*^*p* < 0.001 for baseline vs. FU2

Abbreviations: as in Table 2

### Patients with and without CTRCD

CTRCD occurred at FU2 in 8 of 39 patients (20%) after epirubicin-based chemotherapy and in one of 27 patients (4%) after left-sided radiotherapy, resulting in a total number of 9 of 66 patients (14%) with CTRCD. High sensitive troponin T and T1 and T2 increased in both groups at FU1 and normalized at FU2 (Table 4). LVEF was reduced at FU2 in patients with CTRCD according to the definition of this condition, whereas all strain parameters were already reduced at FU1 and remained reduced at FU2. LV and RV volume reduction typically occurred in both patient groups at FU1 and mostly normalized at FU2.

**Table 4:** Clinical and CMR parameters in patients with and without cancer therapy–related cardiac dysfunction (CTRCD)

	With CTRCD (*n* = 9)				Without CTRCD (*n* = 57)			
	Baseline	FU1	FU2	*p*	Baseline	FU1	FU2	*p*
HS Troponin T, pg/ml	5 ± 3	8 ± 5	10 ± 16	0.60	5 ± 4	7 ± 4^†^	6 ± 6	0.12
NTpro-BNP, pg/ml	148 ± 159	225 ± 537	168 ± 254	0.60	102 ± 95	101 ± 107	121 ± 137	0.04
Hematocrit, %	35 ± 4	34 ± 3	35 ± 4	0.34	38 ± 3	36 ± 4^†^	38 ± 2	< 0.001
Hb, mg/dl	11 ± 1	11 ± 1	12 ± 1	0.19	13 ± 1	12 ± 1^†^	13 ± 1	0.002
Creatinine, mg/dl	0.87 ± 0.28	0.80 ± 0.14	0.86 ± 0.08	0.51	0.79 ± 0.16	0.76 ± 0.11	0.80 ± 0.12	0.04
T1, ms	1246 ± 25	1299 ± 33^‡^	1246 ± 36	< 0.001	1241 ± 29	1266 ± 47^‡^	1246 ± 32	< 0.001
T2, ms	46 ± 5	48 ± 2	46 ± 4	0.17	46 ± 3	47 ± 3^‡^	46 ± 3	0.02
ECV, %	30 ± 2	29 ± 2	0.71	29 ± 3	29 ± 2	0.41		
GLS, %	− 20 ± 1	− 17 ± 3^‡^	− 17 ± 1^#^	0.91	− 18 ± 2	− 18 ± 2	− 18 ± 2	0.29
GCS, %	− 19 ± 2	− 16 ± 3^†^	− 16 ± 3^¶^	0.92	− 18 ± 2	− 18 ± 2	− 18 ± 3	0.33
GRS, %	40 ± 7	31 ± 9^†^	32 ± 5^¶^	0.63	37 ± 7	37 ± 8	37 ± 7	0.90
LVEF, %	61 ± 3	58 ± 6	55 ± 4^ll^	0.20	61 ± 5	62 ± 6	62 ± 6	0.34
LV mass index, g/m^2^	50 ± 6	54 ± 9	54 ± 8	0.95	51 ± 5	51 ± 6	51 ± 6	0.73
LVEDVi, ml/m^2^	79 ± 9	73 ± 11	80 ± 13	0.09	76 ± 10	73 ± 12^‡^	74 ± 11^ll^	0.31
LVESVi, ml/m^2^	31 ± 3	31 ± 7	36 ± 6^ll^	0.03	30 ± 6	27 ± 7^†^	28 ± 7	0.12
LVSVi, ml/m^2^	48 ± 7	43 ± 8	45 ± 8	0.49	47 ± 7	45 ± 9	45 ± 7	0.84
LAESVi, ml/m^2^	36 ± 12	29 ± 6	32 ± 9	0.45	35 ± 10	32 ± 10	34 ± 10	0.10
LAEDVi, ml/m^2^	15 ± 7	14 ± 3	15 ± 4	0.77	15 ± 6	15 ± 6	15 ± 6	0.22
RVEF, %	57 ± 8	54 ± 12	57 ± 6	0.43	56 ± 7	59 ± 7*	59 ± 7^ll^	0.95
RVEDVi, ml/m^2^	78 ± 12	68 ± 15^†^	72 ± 10	0.22	79 ± 11	75 ± 14^†^	75 ± 15^ll^	0.85
RVESVi, ml/m^2^	34 ± 10	32 ± 13	30 ± 5	0.61	35 ± 7	31 ± 7^‡^	31 ± 7^#^	0.99
RVSVi, ml/ m^2^	44 ± 7	36 ± 8*	41 ± 8	0.11	44 ± 8	44 ± 9	44 ± 11	0.82
RAESVi, ml/m^2^	32 ± 7	28 ± 6	32 ± 8	0.07	37 ± 11	34 ± 12*	37 ± 13	0.02
RAEDVi, ml/m^2^	19 ± 6	15 ± 4*	17 ± 7	0.25	21 ± 7	18 ± 7^†^	18 ± 9^ll^	0.50

^*^p < 0.05, ^†^*p* < 0.01 and ^‡^*p* < 0.001 for baseline vs FU1

^ll^*p* < 0.05, ^¶^*p* < 0.01 and ^#^*p* < 0.001 for baseline vs FU2

CTRCD was defined as decline in LVEF of at least 10% to < 55% or a > 15% GLS change at FU2

### Diagnostic accuracy of parameters at FU1 to predict later CTRCD at FU2

**Fig. 4:**
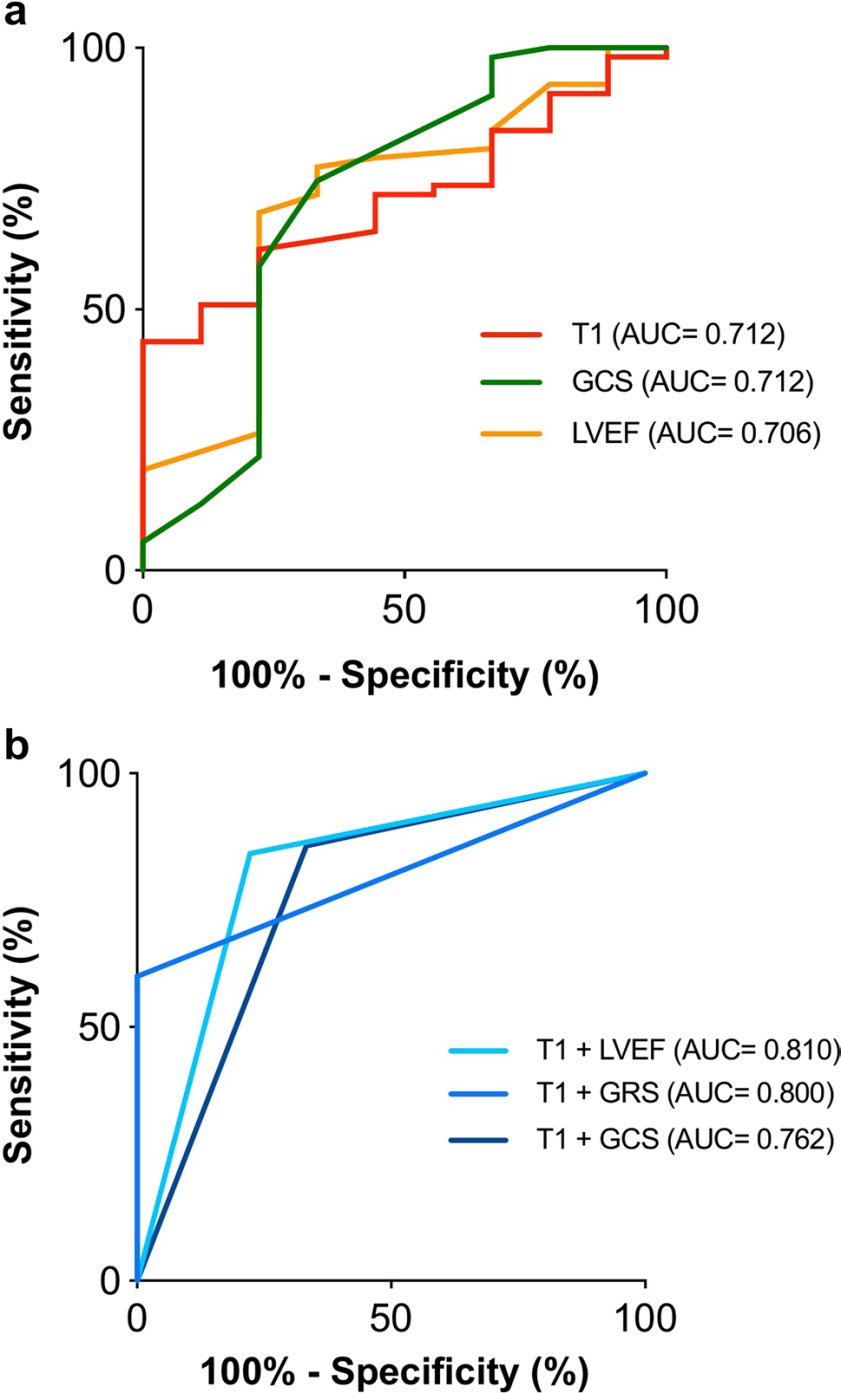
Diagnostic accuracy of parameters at follow-up 1 (FU1) to predict cancer therapy-related cardiac dysfunction (CTRCD) at follow-up 2 (FU2). T1 and global circumferential strain (GCS) at FU1 were the best single parameters to predict CTRCD at FU2 with an AUC of 0.712 (*p* < 0.01) and AUC of 0.712 (*p* < 0.05), respectively (**a**). The combined use of T1 with either left ventricular ejection fraction (LVEF) or global radial strain (GRS) resulted in an improved AUC of 0.810 or 0.800, respectively (**b**)

ROC analysis revealed that T1 and GCS at FU1 were the best single parameters to predict CTRCD at FU2 with an AUC of 0.712 (*p* = 0.005) and 0.712 (*p* = 0.07), respectively (Table 5, Fig. 4a). T1 resulted in excellent sensitivity of 100% (66–100%), but low specificity of 44% (31–58%). Conversely, GCS resulted in an acceptable sensitivity of 67% (30–93%) and specificity of 75% (61–85%). Interestingly, blood parameters of myocardial injury such as high sensitive troponin T and NT-proBNP had low AUC (Table 5). T2 had the lowest AUC of 0.513 of all parameters to predict CTRCD. The combined use of T1 as a parameter with excellent sensitivity with a parameter with higher specificity revealed that the combined use of T1 > 1262 ms and reduced LVEF ≤ 60% following breast cancer treatment at FU1 resulted in an improved AUC = 0.810. This combined use resulted in high sensitivity of 78% (44–95%) and high specificity of 84% (72–92%). The combined use of T1 and GRS or T1 and GCS also improved the AUC to 0.800 and 0.762, respectively. Other combinations of parameters were inferior to the single use of T1 (Table 5, Fig. 4).

**Table 5:** ROC analysis of clinical and CMR parameters at FU1 to predict later CTRCD at FU2

		*p* vs T1				CTRCD (*n* = 9)	No CTRCD (*n* = 57)		Total		Sensitivity	Specificity		Accuracy		PPV	NPV	
Parameter	AUC (95% CI)		Cutoff	TP		FN	FP	TN	(*n* = 66)		(%)	(%)		(%)		(%)	(%)	
T1	0.712 (0.587–0.816)	–-	> 1262 ms	9		0	32	25	66		100 (66–100)	44 (31–58)		52 (40–63)		22 (12–37)	100 (84–100)	
GCS	0.712 (0.585–0.818)	0.951	> -17%	6		3	14	41	64		67 (30–93)	75 (61–85)		73 (61–83)		30 (14–52)	93 (81–98)	
LVEF	0.706 (0.581–0.811)	0.956	≤ 60%	7		2	18	39	66		78 (40–97)	68 (55–80)		70 (58–80)		28 (14–48)	95 (83–100)	
GRS	0.671(0.542–0.783)	0.651	≤ 39%	9		0	35	20	64		100 (66–100)	36 (24–50)		45 (34–57)		20 (11–35)	100 (81–100)	
GLS	0.636 (0.507–0.753)	0.518	> -16%	4		5	7	48	64		44 (14–79)	87 (76–95)		81 (70–89)		36 (15–65)	91 (79–96)	
NT-proBNP	0.625 (0.496–0.742)	0.558	≤ 41 ng/l	6		3	14	42	65		67 (35–88)	75 (62–85)		74 (62–83)		30 (14–52)	93 (81–98)	
HS TNT	0.527 (0.399–0.652)	0.064	> 5 pg/ml	6		3	30	25	65		67 (35–88)	46 (34–59)		49 (37–61)		17 (7–32)	90 (73–97)	
T2	0.513 (0.386–0.638)	0.120	> 45 ms	9		0	44	13	66		100 (66–100)	23 (13–36)		33 (23–45)		17 (9–29)	100 (73–100)	
Combined parameter																		
T1 + LVEF	0.810 (0.695–0.896)	0.298	> 1262 ms/ ≤ 60%	7	2		9	48		66	78 (44–95)	84 (72–92)	83 (72–91)		44 (23–67)			96 (86/100)
T1 + GRS	0.800 (0.681–0.890)	0.258	> 1262 ms/ ≤ 39%	9	0		22	33		64	100 (66–100)	60 (47–72)	66 (53–76)		29 (13–47)			100 (88–100)
T1 + GCS	0.762 (0.640–0.859)	0.639	> 1262 ms/ > -17%	6	3		8	48		65	67 (35–88)	86 (74–93)	83 (72–90)		43 (21–67)			94 (83–99)
T1 + GLS	0.686 (0.558–0.796)	0.752	> 1262 ms/ > 16%	4	5		4	51		64	44 (19–73)	93 (82–98)	86 (75–93)		50 (22–78)			91 (80/97)
T1 + NT-proBNP	0.690 (0.564–0.798)	0.766	> 1262 ms/ ≤ 41 pg/ml	5	4		10	46		65	56 (27–81)	82 (70–90)	78 (67–87)		33 (15–59)			92 (81/97)

*AUC*, area under the curve; *C*I, confidence interval; *GCS*, global circumferential strain, GLS, global longitudinal strain, *GRS*, global radial strain; *HS TNT*, high sensitive troponin T; *LVEF*, left ventricular ejection fraction; *FN*, false negative; *FP*, false positive, *NT-proBNP*, N-terminal pro-brain natriuretic peptide; *PPV*, positive predictive value; *NPV*, negative predictive value; *TP*, true positive; *TN*, true negative; *ROC*, receiver operating characteristic

T1 + LVEF =  > increased T1 > 1262 ms and reduced LVEF < 60%

T1 + GRS =  > increased T1 > 1262 ms and reduced GRS ≤ 39%

T1 + GCS =  > increased T1 > 1262 ms and reduced GCS >  − 17%

T1 + GLS =  > increased T1 > 1262 ms and reduced GLS >  − 16%

T1 + NT-proBNP increased T1 > 1262 ms and NT-proBNP ≤ 41 ng/l

## Discussion

This prospective study analyzed T1 and T2 mapping CMR and laboratory changes in women with breast cancer treated either by epirubicin-based chemotherapy or left-sided radiotherapy. Furthermore, the predictive value of CMR and laboratory parameters for early identification of CTRCD were analyzed. The major findings were as follows: (1) T1 and T2 relaxation times increased after epirubicin-based chemotherapy, but not after left-sided radiotherapy. (2) CTRCD occurred at FU2 in 20% of patients after epirubicin-based chemotherapy, but only in 4% after left-sided radiotherapy. (3) Myocardial T1 after therapy completion was the best single parameter to predict CTRCD with an AUC of 0.712 and excellent sensitivity of 100% (66–100%), but low specificity of 44% (31–58%). (4) The combined use of T1 and reduced LVEF ≤ 60% at FU1 improved the AUC to 0.810 resulting in good sensitivity of 78% (44–95%) and improved specificity of 84% (72 − 92%).

### Post-chemotherapy myocardial injury in breast cancer patients

Galán-Arriola et al recently showed in an animal study that increased T2 is the earliest imaging marker of doxorubicin-related cardiotoxicity [8]. T2 increase was related to intracardiomyocyte edema, which occurred as early as 6 weeks after initiation of chemotherapy in pigs. This study showed that the T2 increase was not paralleled by a decrease of ejection fraction and discontinuing chemotherapy upon detection of T2 prolongation halted LV dysfunction development, demonstrating that early T2 prolongation occurred at reversible disease stages [8]. This study also demonstrated that T1 progressively increases after 10 weeks of doxorubicin and is coincided with a progressive ejection fraction decline [8].

Our study in humans confirms and extends the knowledge about the value of T2 and T1 to detect chemotherapy-related myocardial injury. Interestingly, we found a more pronounced increase in T1 compared to T2 in our patients with chemotherapy. This finding is most likely related to the fact that we performed the first follow-up CMR 2 weeks after chemotherapy completion, which was given for 5 months, whereas Galán-Arriola et al performed weekly CMR [8]. We presume that we imaged the patients at a later time point of myocardial injury when T2 had already normalized, but T1 was still elevated. Similar to Galán-Arriola et al, we observed that the T1 increase was paralleled by a LV dysfunction quantified by strain analysis [8].

### Occurrence of CTRCD

The CTRCD frequency of 20% after chemotherapy is well in line with a recent meta-analysis, which reported a cardiotoxicity frequency of 17% with the highest risk in the high-dose chemotherapy subgroup with 19.6% [16]. The occurrence of CTRCD after radiation is currently less well analyzed. Recent studies showed that left-sided radiation results in reduced myocardial strain by echocardiography [17, 18]. However, the incidence of CTRCD was not reported. Currently, several ongoing studies will analyze early detection and prediction of cardiotoxicity after radiation therapy for breast cancer and will provide more precise data about CTRCD [19, 20]. We observed a low frequency of CTRCD after radiation with 4% by using established criteria. The low frequency is potentially related to the low cardiac radiation dose of 2 ± 2 Gy, which was achieved by dedicated three-dimensional treatment plans including tangential beam orientation and deep inspiration breath-hold technique.

### Diagnostic accuracy of parameters at FU1 to predict later CTRCD at FU2

Our study showed that T1 and GCS at FU1 were the best single parameters to predict CTRCD at FU2 with good AUCs of 0.712. T1 was characterized by excellent sensitivity of 100%, but low specificity of 44%. Conversely, GCS resulted in a balanced sensitivity of 67% and specificity of 75%. Interestingly, blood parameters of myocardial injury such as high sensitive troponin T and NT-proBNP had lower AUCs compared to T1 and GCS, indicating that the increase of these laboratory parameters is an epiphenomenon of chemotherapy and radiation, but they have limited value to identify CTRCD. This is in line with a recent meta-analysis on the use of troponins and NT-proBNP for cardiotoxicity prediction after chemotherapy reporting similar sensitivity of 69%, but higher specificity of 87% for troponins and limited predictive value of NT-proBNP [16]. Interestingly, T2 had the lowest AUC of 0.513 to predict CTRCD. In patients with an acute myocardial injury such as myocarditis or myocardial infarction, T1 and T2 are equally good in identifying disease [21, 22]. In contrast, the current data indicated that T2 is of limited value to identify CTRCD. Altaha et al recently reported a significant overlap of temporal changes in CMR mapping parameters between chemotherapy patients and healthy subjects, whereas T1 showed less overlap than T2 [15]. Similarly, we found an T1 and T2 overlap between patients with and without CTRCD. Thus, the low specificity of T1 and low AUC of T2 might be explained by their moderate increase at the chosen time point following oncologic therapy as opposed to the more pronounced changes caused by myocarditis and myocardial infarction. It is possible, that imaging at a different time point during or after cancer therapy may result in a better performance of these parameters to predict CTRCD.

However, the combined use of increased T1 and reduced cardiac function by LVEF ≤ 60% at FU1 increased the diagnostic accuracy and AUC was 0.810. The combination of T1 and LVEF ≤ 60% resulted in balanced sensitivity (78%), specificity (84%), and accuracy (83%).

### Limitations

The sample size is relatively small. However, this prospective and consecutive study adds to the body of evidence on cardiotoxic effects of cancer therapy in humans and evaluates the predictive value of CMR. Second, our findings are confined to the chemotherapy and radiotherapy applied in this study.

## Conclusions

This prospective CMR study revealed that myocardial T1 and T2 relaxation times increase after epirubicin-based chemotherapy in breast cancer patients, but not after left-sided radiotherapy, indicating the presence of chemotherapy-induced myocardial toxicity. Follow-up 2 CMR at 13 months revealed that 20% of chemotherapy patients developed CTRCD supporting the notion that the observed myocardial injury was associated with LV dysfunction. Conversely, left-sided radiotherapy did not result in increased T1 or T2 and only a small fraction of patients (4%) developed CTRCD later on. ROC analysis revealed that myocardial T1 obtained after therapy completion was the best single parameter to predict CTRCD with an AUC of 0.712 and excellent sensitivity (100%), but low specificity (44%). The combined use of T1 and reduced LVEF ≤ 60% at FU1 improved the AUC to 0.810 resulting in balanced sensitivity (78%), specificity (84%), and accuracy (83%). Therefore, functional and mapping analysis by CMR enables monitoring of chemotherapy-related cardiotoxicity and CTRCD prediction.

## Supplementary Information

Below is the link to the electronic supplementary material.

Supplementary file1 (DOCX 58 KB)

## Abbreviations

AUC: Area under the curve
BMI: Body mass index
BSA: Body surface area
CMR: Cardiovascular magnetic resonance imaging
CTRCD: Cancer therapy-related cardiac dysfunction
EDV: End-diastolic volume
EF: Ejection fraction
ESV: End-systolic volume
GCS: Global circumferential strain
GLS: Global longitudinal strain
GRS: Global radial strain
LA: Left atrial
LV: Left ventricle
RA: Right atrial
ROC: Receiver-operating characteristic
RV: Right ventricle
SV: Stroke volume

## Declarations

### Guarantor

The scientific guarantor of this publication is Enver Tahir, MD.

### Statistics and biometry

Gerhard Schön from the Institute for Medical Biometry and Epidemiology performed the statistical analyses for this paper. He is one of the authors.

### Methodology

• prospective

• longitudinal study

• performed at one institution
