# Supplementary material for: Myocardial injury detected by T1 and T2 mapping on CMR predicts subsequent cancer therapy–related cardiac dysfunction in patients with breast cancer treated by epirubicin-based chemotherapy or left-sided RT
Source: Eur Radiol. 2021 Sep 18;32(3):1853–65. doi: 10.1007/s00330-021-08260-7 (PMC8831341; doi:10.1007/s00330-021-08260-7)
Supplement: Supplementary file 1 — Supplementary file1 (DOCX 58 KB) [file 330_2021_8260_MOESM1_ESM.docx]

**Supplementary Material**

**Methods**

*CMR protocol*

CMR was performed using a 3.0 T MR scanner equipped with a phased array surface receive coil (Ingenia, Philips Healthcare, Best, The Netherlands).

*Cine CMR – steady-state free-precession sequence*

The CMR protocol included standard steady-state free-precession cine CMR in short axis for LV and right ventricle (RV) volumetry and LV mass with the following typical imaging parameters: acquired voxel size (AVS) 2 x 2 x 8 mm^3^, reconstructed voxel size (RVS) 0.99 x 0.99 x 8 mm^3^, no gap, 9-10 slices for full LV coverage, echo time = 1.45 ms, time to repetition = 2.90 ms, flip angle = 45°, parallel acquisition technique = SENSE, acceleration factor 2.

*T1 Mapping – MOLLI sequence*

T1 mapping was performed using a Modified Look Locker Inversion Recovery (MOLLI) sequence with a 5s(3s)3s scheme on three short-axes slices (apical, mid and basal) before and 15 minutes after contrast-media administration as described before (1,2). Typical imaging parameters were as follows: Voxel size 2 x 2 x 10 mm^3^, echo time = 0.7 ms, time to repetition = 2.3 ms, partial echo factor = 0.8, flip angle = 35°, SENSE factor = 2, linear phase encoding, ten start-up cycles to approach steady-state prior to imaging, effective inversion times between 134 and 5627 ms.

*T2-mapping – GraSE sequence*

T2 mapping was performed before administration of contrast-media on three end-diastolic LV short-axes at identical locations as used for T1 mapping, using a gradient- (echo planar imaging) and spin-echo multi-echo sequence (GraSE) in three short-axis sections (3-5)*.* Typical imaging parameters of the GraSE sequence were: Voxel size 2 x 2 x 8 mm^3^, 3 slices, nine echoes with effective echo times between 10.7 and 96.3 ms, time to repetition = 800 ms (1 RR interval), one breath-hold per slice.

*LGE imaging – phase-sensitive inversion recovery – PSIR sequence*

Ten minutes after bolus injection of 0.15 mmol/kg gadoter acid (Dotarem®, Guerbet, Sulzbach, Germany) at a rate of 2.5 ml/s, end-diastolic LGE images were acquired in end-diastole using a phase-sensitive inversion recovery (PSIR) sequence: AVS 1.6 x 1.9 x 8 mm^3^, RVS 0.91 x 0.91 x 8 mm^3^, gap 2 mm, 9-10 slices, echo time = 3 ms, time to repetition = 6.10 ms, flip angle = 25°. The optimal inversion delay was obtained from a Look-Locker experiment. LGE images were acquired in short-axis orientation covering the entire heart and in two-, three- and four-chamber views.

*CMR data analysis*

Two investigators (with 4 and 3 years of experience in CMR, respectively) independently and blindly analyzed each CMR using CVi42 software (Circle Cardiovascular Imaging Inc, Calgary, Alberta, Canada). CMR parameters were indexed to the calculated body surface area (BSA) and are given as the mean of the two observers. Evaluation of LV and RV volumes and LV mass was performed in standard fashion on the short-axis cine stack (6). The epicardial and endocardial borders of the myocardium were manually traced on end-diastolic and end-systolic images at each anatomic level encompassing the entire heart from apex to base. Trabeculae and papillary muscles were included in the ventricular volumes and excluded from the myocardial mass for reproducibility (7). LV mass was calculated by multiplying the myocardial volume by the specific weight of cardiac muscle (1.05 mg/mL) (6). Native T1, post contrast T1 and ECV were obtained by averaging measures from basal and midventricular short-axis slices to yield final measurements (8). Apical slices were not included due to concerns about error related to partial volume averaging (9). To measure global native T1 and T2 relaxation times and ECV corresponding short axis maps were used to carefully delineate endo- and epicardial contours with 10% endo- and epicardial offsets to avoid contamination by blood pool or epicardial tissue (10). ECV was calculated using the previously established equation (11-15).

ECV = 1-hematocrit*R1*_myocardium_*/R1*_blood pool_*

In this equation, R1 is defined as 1/T1 and as the difference between pre- and late post-contrast R1 values (11-15). Hematocrit was measured from a venous blood sample taken immediately before the CMR study from an antecubital vein after 5 min in supine position.

**References Data Supplement**

1. Kellman P, Hansen MS. T1-mapping in the heart: accuracy and precision. J Cardiovasc Magn Reson 2014;16:2.

2. Bohnen S, Prussner L, Vettorazzi E et al. Stress T1-mapping cardiovascular magnetic resonance imaging and inducible myocardial ischemia. Clin Res Cardiol 2019;108:909-920.

3. Baessler B, Schaarschmidt F, Stehning C, Schnackenburg B, Maintz D, Bunck AC. A systematic evaluation of three different cardiac T2-mapping sequences at 1.5 and 3T in healthy volunteers. Eur J Radiol 2015;84:2161-70.

4. Fernandez-Jimenez R, Sanchez-Gonzalez J, Aguero J et al. Fast T2 gradient-spin-echo (T2-GraSE) mapping for myocardial edema quantification: first in vivo validation in a porcine model of ischemia/reperfusion. J Cardiovasc Magn Reson 2015;17:92.

5. Fehrmann A, Treutlein M, Rudolph T et al. Myocardial T1 and T2 mapping in severe aortic stenosis: Potential novel insights into the pathophysiology of myocardial remodelling. Eur J Radiol 2018;107:76-83.

6. Schulz-Menger J, Bluemke DA, Bremerich J et al. Standardized image interpretation and post-processing in cardiovascular magnetic resonance - 2020 update : Society for Cardiovascular Magnetic Resonance (SCMR): Board of Trustees Task Force on Standardized Post-Processing. J Cardiovasc Magn Reson 2020;22:19.

7. Papavassiliu T, Kuhl HP, Schroder M et al. Effect of endocardial trabeculae on left ventricular measurements and measurement reproducibility at cardiovascular MR imaging. Radiology 2005;236:57-64.

8. Schelbert EB, Piehler KM, Zareba KM et al. Myocardial Fibrosis Quantified by Extracellular Volume Is Associated With Subsequent Hospitalization for Heart Failure, Death, or Both Across the Spectrum of Ejection Fraction and Heart Failure Stage. J Am Heart Assoc 2015;4.

9. Moon JC, Messroghli DR, Kellman P et al. Myocardial T1 mapping and extracellular volume quantification: a Society for Cardiovascular Magnetic Resonance (SCMR) and CMR Working Group of the European Society of Cardiology consensus statement. J Cardiovasc Magn Reson 2013;15:92.

10. Bohnen S, Avanesov M, Jagodzinski A et al. Cardiovascular magnetic resonance imaging in the prospective, population-based, Hamburg City Health cohort study: objectives and design. J Cardiovasc Magn Reson 2018;20:68.

11. Ugander M, Oki AJ, Hsu LY et al. Extracellular volume imaging by magnetic resonance imaging provides insights into overt and sub-clinical myocardial pathology. Eur Heart J 2012;33:1268-78.

12. Arheden H, Saeed M, Higgins CB et al. Measurement of the distribution volume of gadopentetate dimeglumine at echo-planar MR imaging to quantify myocardial infarction: comparison with 99mTc-DTPA autoradiography in rats. Radiology 1999;211:698-708.

13. Jerosch-Herold M, Sheridan DC, Kushner JD et al. Cardiac magnetic resonance imaging of myocardial contrast uptake and blood flow in patients affected with idiopathic or familial dilated cardiomyopathy. Am J Physiol Heart Circ Physiol 2008;295:H1234-H1242.

14. Schelbert EB, Testa SM, Meier CG et al. Myocardial extravascular extracellular volume fraction measurement by gadolinium cardiovascular magnetic resonance in humans: slow infusion versus bolus. J Cardiovasc Magn Reson 2011;13:16.

15. Kellman P, Wilson JR, Xue H, Ugander M, Arai AE. Extracellular volume fraction mapping in the myocardium, part 1: evaluation of an automated method. J Cardiovasc Magn Reson 2012;14:63.

**Table S1:** No indication was present, that trastuzumab treatment had additional cardiotoxic effects since this data revealed no differences between laboratory or mapping parameters, ejection fraction and myocardial strain in group 1 patients without (n=34) and with (n=5) trastuzumab treatment at all obtained measurements.

**Baseline Follow-up 1 Follow-up 2**

(N=34) (N=5) *P* (N=34) (N=5) *P* (N=33) (N=5) *P*

HS Troponin T, pg/ml 5 ±5 5 ±1 0.68 8 ±5 8 ±4 0.87 8 ±11 7 ±3 0.81

NTpro-BNP, pg/ml 108 ±106 207 ±163 0.08 145 ±293 63 ±50 0.54 146 ±188 119 ±86 0.76

Hematocrit, % 37 ±4 37 ±2 0.95 35 ±4 34 ±5 0.85 38 ±3 36 ±1 0.17

Hb, mg/dl 13 ±1 13 ±1 0.60 12 ±1 12 ±2 0.79 13 ±1 12 ±1 0.34

Creatinine, mg/dl 0.83 ±0.21 0.75 ±0.08 0.37 0.77 ±0.14 0.72 ±0.1 0.50 0.84 ±0.12 0.77 ±0.1 0.20

T1, ms 1245±28 1239±36 0.66 1294 ±36 1286 ±24 0.65 1248 ±27 1266 ±14 0.16

T2, ms 45 ±3 46 ±2 0.42 48 ±3 47 ±4 0.40 46 ±3 46 ±3 0.89

ECV, % 28 ±2 29 ±2 0.71 29 ±2 30 ±2 0.85

GLS, % -18 ±2 -19 ±2 0.75 -17 ±2 -18 ±2 0.33 -17 ±2 -18 ±2 0.15

GCS, % -18 ±2 -19 ±3 0.31 -17 ±3 -18 ±2 0.99 -17 ±3 -18 ±1 0.37

GRS, % 36 ±7 32 ±7 0.16 34 ±8 34 ±4 0.87 34 ±6 34 ±7 0.87

LVEF, % 60 ±4 64 ±7 0.08 60 ±6 60 ±4 0.99 59 ±7 62 ±4 0.44

RVEF, % 56 ±8 59 ±4 0.35 59 ±8 58 ±4 0.98 58 ±6 58 ±9 0.94
